# Supplementary material for: WordCommentsAnalyzer: A windows software tool for qualitative research
Source: F1000Res. 2018 Sep 4;7:536. Originally published 2018 May 3. [Version 2] doi: 10.12688/f1000research.14819.2 (PMC6137411; doi:10.12688/f1000research.14819.2)
Supplement: Supplementary file 3 [file f1000research-7-17614-s0002.tgz › b2a79496-4ada-47c4-9682-a99ce867836d.zip!/Eight Iranian Health Journals/Archives of Rehabilitation_51.docx]

© Archives of Rehabilitation. This work is licensed under CC BY-NC 4.0 (https://creativecommons.org/licenses/by-nc/4.0/).

Volume & Issue: دوره 8 - شماره 1

Title: بررسی عوامل خطر اقدام به خودکشی در شهرستان اردبیل در نیمه اول سال 1382

http://rehabilitationj.uswr.ac.ir/article-1-15-fa.html

هدف: خودکشی از نظر مفهومی عمل خاتمه دادن عمدی به زندگی خود است به شرط اینکه به میل خود شخص و به دست خود او انجام پذیرد. خودکشی یک معضل عمده در بهداشت و سلامت اجتماعی می‌باشد و میزان آن در بین افراد 24-15ساله درحال افزایش است. این مطالعه با هدف شناسایی عوامل خطر و زمینه‌ساز عمده و اساسی در خودکشی انجام شده است. روش بررسی: پژوهش انجام شده یک مطالعه توصیفی - مقطعی است. واحد آماری در مطالعه حاضر افرادی بوده‌اند که در نیمسال اول 1382 اقدام به خودکشی کرده و در بیمارستانهای فاطمی و بوعلی شهرستان اردبیل بستری شده‌اند. حجم نمونه ۲۱۸ مورد بوده و در هر رده سنی و از هر دو جنس بوده است که با روش تمام شماری همگی در مطالعه شرکت داده شدند. مصاحبه بالینی بوسیله پرسشنامه محقق ساخته با بیماران و بستگان درجه اول آنان و آزمون اجرا شده MMPI ابزار تحقیق بوده است. سپس داده‌های مطالعه مورد تجزیه و تحلیل آماری قرار گرفته است. یافته‌ها: در پژوهش انجام شده بالاترین موارد اقدام به خودکشی (درصد) در رده سنی 24-15 ساله، در جنس مؤنث (۶۱درصد)، در افراد متأهل (53/22 درصد)، با تحصیلات در حد دبیرستان تا دیپلم (35/78 درصد) و در طبقه اقتصادی – اجتماعی متوسط (57/34 درصد) بوده است. 61/47 درصد این افراد دچار اختلالات روانپزشکی و 57/72 درصد دچار اختلالات شخصیتی بوده‌اند. 47/77 درصد این افراد از حمایت اجتماعی مؤثر برخوردار نبوده‌اند. بیشترین روش به کار رفته جهت اقدام به خودکشی استفاده از داروها و سموم بوده است (90/83 درصد) و بیشترین علت خودکشی اختلاف با همسر گزارش شده است. نتیجه‌گیری: این بررسی نشان دهنده فراوانی و ارتباط عوامل خطر مختلفی است که به نحوی در اقدام به خودکشی نقش دارند و شامل شرایط اجتماعی – اقتصادی متوسط، تحصیلات پایین، اواخر نوجوانی و ابتدای جوانی، زن بودن، متأهل بودن، اختلافات خانوادگی بویژه بین زوجین تازه ازدواج کرده و اختلالات روانپزشکی و شخصیتی و دستیابی آسان به داروها و سموم می‌باشد و با توجه به نیمرخ روانشناختی، این افراد از نظر تفکر دارای سوء ظن و بدبینی، سوء تعبیر محرکها، اشتغال فکری زیاد و میل به گوشه‌گیری هستند.

Title: شیوع علائم و نشانه‌های وسواسی – جبری و برخی عوامل جمعیت شناسی مرتبط با آن در دانش‌آموزان دوره راهنمایی شهر تهران سال 1384

http://rehabilitationj.uswr.ac.ir/article-1-14-fa.html

هدف: نظر به اهمیت بهداشت روان و مدارس بعنوان جایگاهی مناسب برای اشاعه و ارتقاء آن، و از آنجائیکه اولین گام در این راه بررسی وضعیت موجود است، لذا هدف این مطالعه بررسی شیوع یکی از مؤلفه‌های مهم بهداشت روان در سنین نوجوانی تحت عنوان اختلال وسواسی – جبری و تعدادی از عوامل مرتبط با آن از جمله سن، جنس، سطح تحصیلات پدر و مادر، تعداد خواهر و برادر و ترتیب تولد نوجوان در خانواده و همراهی این اختلال با دیگر اختلالات شایع روانی می‌باشد. روش بررسی: در این مطالعه مقطعی، 672 دانش‌آموز دوره راهنمایی، 369 دختر و 303 پسر، محصل در مدارس شهر تهران بررسی شدند. روش نمونه‌گیری بصورت چند مرحله‌ای و خوشه‌ای با انتخاب چهار منطقه از مناطق شمال، غرب، شرق و جنوب آموزش و پرورش شهر تهران و سپس در هر منطقه دو مدرسه راهنمایی (یکی پسرانه و یکی دخترانه) و در هر مدرسه حداقل دو مقطع مختلف تحصیلی صورت گرفت. داده‌ها توسط پاسخ به پرسشنامه SCL-90R توسط کلیه دانش‌آموزان حاضر در کلاسهای انتخاب شده، جمع‌آوری شد. یافته‌ها: با احتساب میانگین نمرات کسب شده از سئوالات کلیدی مربوط به علائم وسواسی - جبری در هر فرد، درصد افرادی که نمره 2 و بالاتر کسب کرده‌اند با حدود اطمینان 95% (%13/2, %8/3) بدست آمد که در دختران %12/3 و در پسران %8/7 است که البته این اختلاف از نظر آماری معنی‌دار نیست (P=0/16). از بین عوامل مرتبط نیز سن و منطقه جغرافیایی مدرسه با شیوع علائم ارتباط معنی‌دار آماری دارند به‌گونه‌ای که با افزایش سن، شیوع این اختلال افزایش می‌یابد. از بین مناطق جغرافیایی نیز منطقه شرق و جنوب آموزش و پرورش درصد بیشتری از علائم این اختلال را به خود اختصاص می‌دهند. از بین نشانه‌های همراه نیز، شایعترین، علائم اضطرابی است. سطح معنی‌داری در همه آزمونهای آماری P<0/05 در نظر گرفته شده است. نتیجه‌گیری: با نظر به شیوع بالای این علائم در بررسی انجام شده، توجه به امر بهداشت روان در مدارس بخصوص در زمینه علائم وسواسی – جبری، بیش از پیش روشن می‌شود. لذا آموزش دانش‌آموزان و معلمان آنها و مسئولین مربوطه در آموزش و پرورش و نیز ارزیابی بالینی جهت تشخیص قطعی و در صورت لزوم اقدامات درمانی پیشنهاد می‌شود.

Title: بررسی علل نابینایی و کاهش شدید دید در دانش‌آموزان مدارس نابینایان مشهد در سال 1385

http://rehabilitationj.uswr.ac.ir/article-1-13-fa.html

هدف: تعیین علل کاهش شدید دید و نابینایی و تعیین میزان حدت بینایی در دانش‌آموزان مدارس نابینایان شهر مشهد به منظور ارزیابی علل قابل پیشگیری هدف این مطالعه می‌باشد. روش بررسی: در سال 1385 طی یک مطالعه تحلیلی و با استفاده از پروتکل تعدیل شده سازمان بهداشت جهانی برای بچه‌های نابینا و نیمه بینا 260 دانش‌آموز شاغل به تحصیل در چهار مدرسه نابینایان در مشهد مورد معاینه قرارگرفتند. اطلاعات جمع آوری شده توسط اپتومتریست و متخصص چشم شامل پرسشنامه و معاینات بالینی آنالیزو نقص اصلی تشریحی و علت اصلی بوجود آورنده آن برای هر دو چشم جداگانه مشخص و ثبت گردید. یافته‌ها: در جمعیت مورد مطالعه 157 نفر (%54/9) مذکر و 129 نفر (%45/1) مؤنث بودند. متوسط سن دانش‌آموزان 4/2±14/2 سال بود. بیشترین علل نابینایی مربوط به بیماریهای رتین (%28/6) و بعد از آن به ترتیب آتروفی عصب بینایی، لنز (کاتاراکت مادرزادی)، کدورت قرنیه، گلوکوم مادرزادی، آلبینیسم، میکروفتالموس، آنوفتالموس و ضایعات یووه آ بودند. بیشترین علل جراحی انجام شده کاتاراکت بود و میزان شیوع عدم درک نور در دو جنس از نظر آماری معنی‌دار بود (P<0/01). نتیجه گیری: بیماریهای ارثی نقش مهمی در میان علل نابینایی دارند، بنابراین مشورت ژنتیکی و غربالگری در سنین پایین و آموزش عمومی می‌تواند از بروز نابینایی پیشگیری، یا از شیوع آن کاسته و یا شدت آن را کم نماید.

Title: مقایسه توجه انتخابی شنیداری و توجه تقسیم شده شنیداری در بیماران مبتلا به سکته مغزی با افراد هنجار

http://rehabilitationj.uswr.ac.ir/article-1-7-fa.html

هدف: بیماریهای عروقی مغز از جمله مهمترین و شایعترین بیماریهایی می‌باشند که بر سیستم شنیداری مرکزی اثر می‌گذارند. در پژوهش حاضر سعی گردیده است تا وضعیت توجه شنیداری (انتخابی و تقسیم شده) بیماران مبتلا به سکته مغزی 75-40 ساله که هیچگونه شکایتی از شنوایی خود نداشتند، بررسی شده و با افراد هنجار مقایسه گردد. روش بررسی: مطالعه مقطعی مقایسه‌ای حاضر، روی 15 بیمار مبتلا به سکته مغزی با جنسیت مذکر و رده سنی 75-40 سال که با نمونه‌گیری هدفمند و غیر احتمالی گزینش و 15 فرد هنجار که با بیماران جور شده بودند انجام گرفت. بیماران مذکور به دو گروه تقسیم شدند: 6 بیمار با ضایعه در نیمکره چپ و 9 بیمار با ضایعه در نیمکره راست. سپس به منظور بررسی توجه انتخابی شنیداری و توجه تقسیم شده شنیداری به ترتیب از آزمونهای جملات رقابتی و اعداد دایکوتیک در نسخه‌های اصلاح شده فارسی استفاده گردید. یافته‌ها: میانگین امتیاز آزمون جملات رقابتی در هر دو گوش بیماران مبتلا به سکته مغزی با میانگین امتیاز این آزمون در هر دو گوش افراد هنجار در برخی از موارد، اختلاف معنا داری داشت (P<0/05). میانگین امتیاز آزمون اعداد دایکوتیک در هر دو گوش بیماران مبتلا به سکته مغزی با میانگین امتیاز این آزمون در هر دو گوش افراد هنجار در عمده موارد اختلاف معنا داری داشت (P<0/05). نتیجه‌گیری: بر اساس یافته‌های پژوهش، توجه انتخابی شنیداری در مواردی اندک و در مقابل توجه تقسیم شده شنیداری در عمده موارد تحت تأثیر سکته مغزی قرار می‌گیرند. بیماران مبتلا به سکته مغزی که شواهدی از زبان پریشی در آنها مشاهده نمی‌شود، اختلالاتی در روند پردازش شنیداری مرکزی آنها آشکار می‌گردد. به عبارت دیگر، کم شنوایی مخفی این دسته از بیماران در قالب اختلالات توجه شنیداری نمود می‌یابد.

Title: بررسی تأثیر آنتی اکسیدان ملاتونین بر شکنج‌های حسی-حرکتی مغز موش بعد از القاء ادم مغزی

http://rehabilitationj.uswr.ac.ir/article-1-6-fa.html

هدف: ترکیبات زیادی را می توان نام برد که برای سلولهای عصبی دارای نقش محافظتی هستند. یکی از این مواد ملاتونین است که به عنوان یک آنتی اکسیدان قوی و جمع کننده رادیکالهای آزاد مطرح می‌باشد.هدف این مطالعه بررسی تأثیر ملاتونین بر (کورتکس) کورتکس حسی- حرکتی مغز موش بعد از ایجاد ضایعه مغزی با استفاده از شوک سرمایی بود.  روش بررسی: به منظور دستیابی به این هدف، در یک مطالعه مداخله‌ای و تجربی ملاتونین با دوزهای mg/kg 100 و 50، 5، 1 به صورت داخل صفاقی به موشهایی گروههای آزمون از کل موش‌هایی که بطور تصادفی به 8 گروه تقسیم شده بودند و در ناحیه آهیانه (پاریتال) مغز آنها با استفاده از شوک سرمایی ضایعه ایجاد شده بود تزریق شد. تزریق در سه نوبت (0/5 ساعت قبل، 24 ساعت و 48 ساعت بعد از ایجاد ضایعه) انجام شد. 72 ساعت بعد از ایجاد ضایعه، مغز موش‌ها برداشته شد و برشهایی به منظور مطالعه با میکروسکوپ نوری تهیه و پس از رنگ آمیزی کرزیل ویوله، مطالعات ایمونوهیستوشیمیایی به منظور بررسی آنتی بادی‌های Bax و Survivin در مقاطع بافتی انجام و نهایتاً سلولهای زنده در قشر مغز این موش‌ها شمارش شد. یافته‌ها: نتایج حاصله نشان داد که شوک سرمایی به شکل معنی‌داری باعث کاهش تعداد سلولهای زنده شده است. تجویز ملاتونین بعد از ایجاد ضایعه در گروه‌های مورد مطالعه باعث افزایش تعداد سلولهای زنده شد. افزایش تعداد این سلولها در گروه‌هایی که ملاتونین را با دوزهای پایین (mg/kg 1 و 5) دریافت کردند نسبت به گروه کنترل معنی‌دار نبوده، ولی در گروهی که ملاتونین را با دوزبالا دریافت کردند (mg/kg 50)، افزایش تعداد سلولها نسبت به گروه کنترل معنی‌دار بود (P>0/05). دوز mg/kg 100 سمی بود. بررسی‌های ایمونوهیستوشیمی نشان دادکه مسیرهای مختلف مرگ سلولی (نکروز وآپوپتوز) به دنبال شوک سرمایی ایجاد گردیده است.  نتیجه‌گیری: تجویز ملاتونین با دوز مناسب می تواند سبب کاهش آسیب ناحیه قشری در مغز موش‌هایی که بااستفاده از شوک سرمایی دچار ضایعه مغزی شده اند، گردد و به این ترتیب باعث کاهش مرگ سلولها بعد از ایجاد ضایعه گردد.

Title: بررسی ارتباط بین نتایج آزمون‌های عملکردی و مقادیر ایزوکینتیکی قدرت عضلات اطراف مفصل زانو در بیماران مبتلا به سندرم درد مفصل کشککی ـرانی

http://rehabilitationj.uswr.ac.ir/article-1-5-fa.html

هدف: سندرم درد مفصل کشککی ـ رانی حدود 25درصد از ضایعات مفصل زانو را به خود اختصاص می دهد. این عارضه فعالیت‌های روزمره افراد را تحت تأثیر قرار داده و منجر به ناتوانی‌های عملکردی می‌گردد. هدف این مطالعه بررسی ارتباط نتایج آزمون‌های عملکردی با شاخص‌های ایزوکینتیک قدرت عضلات اطراف مفصل زانو و ارزیابی subjective عملکرد زانو در بیماران مبتلا به درد مفصل کشککی ـ رانی و مقایسه آن با افراد سالم می‌باشد. روش بررسی:‌ این مطالعه تحلیلی مورد – شاهدی بر روی 15 فرد سالم و 15 بیمار مبتلا به سندرم درد مفصل کشککی ـ رانی که بصورت هدفمند انتخاب شده بودند طی دو مرحله اصلی صورت پذیرفت. 1- آزمون‌های عملکردی که هر فرد آزمون‌های عملکردی پایین آمدن از پله و چمباتمه زدن با دو پا را به صورت تصادفی (با محاسبه تعداد تکرار در مدت 30 ثانیه) و با زمان استراحت 3 دقیقه بین هر آزمون انجام می‌داد. سپس پرسشنامه کاجالا جهت ارزیابی subjective عملکرد زانو تکمیل گردید. 2 - آزمون‌های ارزیابی ایزوکینتیکی قدرت عضلات زانو که از دینامومتر بایودکس در دامنه حرکتی 10 تا 90 درجه فلکسیون زانو ودر سرعت‌های 60 و 120 درجه بر ثانیه استفاده شد. یافته‌ها: نتایج حاصل از این مطالعه نشان داد که هیچگونه ارتباط آماری بین آزمون‌های عملکردی و ارزیابی ایزوکینتیک عضلات زانو در افراد بیمار مشاهده نمی‌شود. بین آزمون‌های عملکردی چمباتمه زدن با دو پا و پایین آمدن از پله و ارزیابی subjective عملکرد زانو ارتباط ضعیف معنادار آماری مشاهده گردید (به ترتیب 0/47=r و 0/37=r). بین ارزیابی subjective عملکرد زانو و شاخص ایزوکینتیک نسبت عملکردی قدرت عضله همسترینگ به چهارسررانی در حرکت فلکسیون زانو در سرعت 60 درجه بر ثانیه، ارتباط متوسط و معکوس معنادار مشاهده گردید (0/58-=r). کلیه پارامترهای ایزوکینتیک عضله چهارسررانی در سرعت 60 درجه بر ثانیه و همچنین مقادیر آزمونهای عملکردی و پرسشنامه کاجالا بین افراد سالم و بیمار تفاوت معنادار آماری مشاهده شد. نتیجه‌گیری: بدلیل عدم وجود ارتباط معنادار قوی بین ارزیابی ایزوکینتیک قدرت عضلات اطراف مفصل زانو و آزمونهای عملکردی و ارزیابی subjective عملکرد زانو در بیماران مبتلا به سندرم درد مفصل کشککی ـ رانی، استفاده از این روشهای ارزیابی به جای یکدیگر در این بیماران توصیه نمی‌شود. لذا استفاده از هر سه روش مذکور برای درک بهتر از وضعیت عملکردی و عضلانی این بیماران لازم است.

Title: مقایسه شیوع برتری دستی در بین دانش‌آموزان طبیعی و ناشنوای مادرزاد 18-12 ساله شهر تهران

http://rehabilitationj.uswr.ac.ir/article-1-8-fa.html

هدف: برتری دستی، بارزترین عدم تقارن رفتاری در انسان است. اصطلاح برتری دستی به دست برتر یا به عدم تقارن عملکرد در اعمال دستی اشاره دارد. در مطالعه حاضر، مقایسه شیوع چپ دستی در بین دانش‌آموزان طبیعی و ناشنوا، تأثیر سن، جنسیت، و توصیه به استفاده از دست راست در اعمال تک‌دستی بر برتری دستی، فراوانی افراد چپ‌دست در خانواده دانش‌آموزان، تأثیر برتری دستی بر توانایی زبان انگلیسی و بررسی دیدگاه افراد جامعه نسبت به چپ‌دستان، مد نظر بود. روش بررسی: مطالعه مقطعی- مقایسه‌ای حاضر از طریق تکمیل پرسشنامه، با استفاده از مقیاس برتری دستی ادینبورگ روی 380 نفر دانش‌آموز طبیعی با انتخاب احتمالی و 380 نفر ناشنوای شدید یا عمیق حسی- عصبی مادرزادی با انتخاب غیر احتمالی و هدفمند در محدوده سنی 18-12 سال در شهر تهران صورت گرفت. به جز کم شنوایی در دانش‌آموزان ناشنوا، افراد مورد مطالعه ، هیچ گونه بیماری یا مشکل نورولوژیک تأیید شده‌ای نداشتند. برای تعیین میزان و نوع کم شنوایی، به پرونده وضعیت شنوایی دانش‌آموزان و تأیید شنوایی شناس مدارس ناشنوایان، استناد شد. یافته‌ها: در مطالعه حاضر، شیوع چپ دستی در دانش‌آموزان طبیعی %9/7 و در ناشنوایان %10/3 و بسیار به یکدیگر نزدیک بود، و بین آنها تفاوت معناداری وجود نداشت (P=0/901, z=-1/24). در هر دو گروه دانش‌آموزان طبیعی و ناشنوا، اگرچه شیوع چپ دستی در پسران بیش از دختران بود، اما جنسیت و سن بر نتایج به دست آمده تأثیری نداشت (P>0/05). توصیه خانواده و یا معلمان به استفاده از دست راست، در دانش‌آموزان طبیعی 16 درصد و در دانش‌آموزان ناشنوا 0/5 درصد بود و هیچ‌گونه توصیه اکید یا سخت‌گیری در این زمینه، اعمال نشده بود. فراوانی افراد چپ دست، در خانواده دانش‌آموزان طبیعی (%22/6) نسبت به دانش‌آموزان ناشنوا (%13/2) بالاتر و این اختلاف از لحاظ آماری معنادار بود (P=0/003, Z=-2/976). در هر دو گروه دانش‌آموزان طبیعی و ناشنوا، بین راست دستان و چپ دستان در امتیازات زبان انگلیسی، تفاوت معناداری وجود نداشت (P>0/05). همچنین، در بررسی دیدگاه افراد جامعه نسبت به چپ دستان، بین دو گروه دانش‌آموزان طبیعی و ناشنوا، تفاوت معناداری مشاهده نشد (P=0/541, Z=-0/611). نتیجه‌گیری: شیوع چپ دستی در بررسی حاضر، به شیوع آن در آمریکای شمالی و اروپای غربی نزدیک بود و با آمار برخی کشورهای آسیایی تفاوت قابل توجهی را نشان داد. مشابهت مقادیر شیوع چپ دستی در دانش‌آموزان طبیعی و ناشنوا، فرضیه پرویک در خصوص عدم شکل گیری «برتری گوش راست» در ناشنوایان مادرزاد را تأیید نکرد.

Title: بررسی تأثیر افت شنوایی بر مهارت‌های سریع حرکتی دهانی، سرعت گفتار و قابلیت فهم گفتار

http://rehabilitationj.uswr.ac.ir/article-1-9-fa.html

هدف: تأثیر منفی افت شنوایی بر مهارتهای حرکتی سریع اندام‌های گفتاری، سرعت گفتار و قابلیت فهم گفتار هر یک به تنهایی در افراد کم‌شنوا به اثبات رسیده است. بررسی نحوه این تأثیرگذاری در آستانه‌های مختلف شنوایی و چگونگی وجود ارتباط بین این سه متغیر گفتاری و ارتباط بین آزمون‌های موجود برای بررسی این متغیرها هدف این پژوهش می‌باشد. روش بررسی: این مطالعه تحلیلی مقطعی بر روی 46 بزرگسال مبتلا به افت شنوایی (عمیق، شدید، متوسط- شدید) و 15 بزرگسال با شنوایی طبیعی انجام شده است. آزمودنیها به صورت تصادفی و از دبیرستانهای مخصوص افراد دچار افت شنوایی و دبیرستانهای طبیعی تهران انتخاب شدند. آزمون‌های مورد استفاده شامل بخشی از آزمون دیادوکوکینزیس دهانی فلچر که با تحلیل‌گر گفتاری دکتر اسپیچ نمونه‌های ضبط شده تحلیل می‌شوند، متن صد کلمه‌ای (برای سنجش سرعت گفتار بر حسب تعداد کلمات خوانده شده در دقیقه) و استفاده از روش شاخص رتبه‌ای برای تعیین رتبه قابلیت فهم گفتاری آزمودنی‌ها در خواندن متن بودند. سپس داده‌ها مورد تجزیه و تحلیل آماری قرار گرفت. یافته‌ها: نتایج کلی حاکی از آن بود که با افزایش آستانه شنوایی، آزمودنیها مدت زمان بیشتری را صرف انجام تکالیف دیادوکوکینزیس دهانی کردند و از سرعت و قابلیت فهم گفتار آنها نیز کاسته شد. البته در مورد سرعت گفتار و توانایی دیادوکوکینزیس دهانی بین خود گروه‌های مبتلا به افت شنوایی، فقط تفاوت بین گروه مبتلا به افت شنوایی عمیق با دو گروه شدید و متوسط-شدید معنی‌دار بود (P<0/05). در ضمن در گروه مبتلا به افت شنوایی بین سرعت گفتار و مدت زمان صرف‌شده برای تکرار تکالیف دیادوکوکینزیس دهانی ارتباط منفی و معنی‌داری وجود داشت (P=0/02). اما در گروه شنوایی طبیعی هیچ گونه ارتباط معنی‌داری بین این دو مشاهده نشد (P>0/05). کلاً بین دیادوکوکینزیس دهانی و قابلیت فهم گفتار و همچنین سرعت گفتار و قابلیت فهم گفتار نیز ارتباط معنی‌داری مشاهده شد (P>0/05). نتیجه‌گیری: با توجه به تأثیر منفی افت شنوایی بر مهارت‌های سریع حرکتی دهانی، سرعت گفتار وقابلیت فهم گفتار و وجود ارتباط بین این سه متغیر، در مداخلات درمانی بر روی افراد دچار افت شنوایی، شاید بتوان به عنوان مثال با مداخله و بهبود دادن مهارتهای سریع حرکتی دهانی مانند تواناییهای دیادوکوکینزیس دهانی باعث بهبود بخشیدن به سرعت گفتار و یا حتی قابلیت فهم گفتار آنها شد.

Title: ترجمه و بومی‌سازی پرسشنامه پیامد صدمات زانو و استئوآرتریت (KOOS) و بررسی تکرار پذیری نسخه فارسی در ایرانیان مبتلا به استئوآرتریت زانو

http://rehabilitationj.uswr.ac.ir/article-1-10-fa.html

هدف: دستیابی به یک وسیله ارزیابی تکرار پذیر جهت بررسی کیفیت زندگی ایرانیان مبتلا به استئوآرتریت از طریق ترجمه و بومی‌سازی پرسشنامه پیامد صدمات زانو و استئوآرتریت (KOOS) و بررسی تکرارپذیری آن در ایرانیان مبتلا به استئوآرتریت هدف این مطالعه است. روش بررسی: تحقیق حاضر یک مطالعه غیر تجربی از نوع متدولوژیک می باشد. پرسشنامه KOOS طی سه مرحله طبق روش IQOLA به زبان فارسی ترجمه و بومی‌سازی شد و برای بررسی تکرارپذیری، در دو نوبت با فاصله حداکثر یک هفته در اختیار 30 نفر فارسی زبان که طبق تشخیص پزشک مبتلا به استئوآرتریت زانو بودند و با دستور فیزیوتراپی زانوها به کلینیک‌های شهرداری و کلینیک سهروردی مراجعه کرده بودند قرار گرفت. روش نمونه گیری به صورت ساده و غیر احتمالی بود. سپس داده‌های بدست ‌آمده از لحاظ تکرار‌پذیری مطلق و نسبی در دفعات آزمون و همخوانی درونی خرده مقیاس‌ها و همخوانی درونی اعتبار افتراقی در سطح آیتم‌ها، تحت تجزیه و تحلیل آماری قرار گرفت. یافته‌ها: همخوانی درونی هر یک از خرده مقیاس‌های پرسشنامه KOOS که توسط ضریب آلفای کرونباخ تخمین زده شده بود به جز در خرده مقیاس علائم که در حد متوسطی قرارداشت، در سایر زیر مجموعه‌ها بالا بود (حداقل 0/76) و نشان‌می‌داد که آیتم‌های هر خرده مقیاس یک مفهوم را ارزیابی می‌کنند. همبستگی هر آیتم با خرده مقیاس خود، بعد از اصلاح همپوشانی، برای آیتم‌های همه خرده مقیاس‌ها به جز آیتم‌های خرده مقیاس علائم بالاتر از حد مطلوب 0/4 بود که همخوانی درونی خوبی را در سطح آیتم‌های این خرده مقیاس‌ها نشان می‌داد. شاخص خطای معیار اندازه‌گیری و ضریب ICC که به ترتیب برای بررسی تکرارپذیری مطلق و نسبی نسخه فارسی پرسشنامه KOOS در دفعات آزمون به کار رفته بودند، تکرارپذیری خوب نسخه فارسی پرسشنامه KOOS را نشان دادند که از مقدار قابل قبول 0/7 بالاتر بود و حداکثر شاخص خطای معیار اندازه‌‌گیری 7/44 بود که از حداقل تفاوت قابل درک برای بیمار، که برای پرسشنامه KOOS 8 تا 10 محاسبه شده کمتر بود.  نتیجه‌گیری: با تهیه نسخه فارسی پرسشنامه KOOS یک وسیله ارزیابی چند منظوره در اختیار است که می‌توان با استفاده از آن وضعیت سلامتی و تأثیر درمانهای مختلف را روی بیماران مبتلا به استئوآرتریت زانو در کشورمان بررسی نمود. در ضمن برای بهینه‌سازی پرسشنامه و تناسب بیشتر آن برای ارزیابی بیماران باید خرده مقیاس علائم را مورد اصلاح قرار داد.

Title: خواص روانسنجانه مقیاس سنجش نگرش نوجوانان به اعتیاد

http://rehabilitationj.uswr.ac.ir/article-1-11-fa.html

هدف: اصلاح نگرش افراد به اعتیاد یکی از راههای شناخته شده پیشگیری از اعتیاد است. به منظور سنجش نگرش نوجوانان به اعتیاد، معتاد، و مواد ؛ و برای ارزیابی عینی روند تغییر آن بر اثر مداخلات گوناگون پیشگیرانه، مقیاسی تهیه و خواص روانسنجانه (سایکومتریک) آن بررسی شد. روش بررسی: این پژوهش به شیوه تحلیلی و در دو مرحله تهیه گویه ها و اجرای آزمایشی مقیاس صورت گرفت. در مرحله اجرا 80 دانش آموز دختر و پسر در محدوده سنی 11 تا 16 سال مورد بررسی قرار گرفتند. پس از تکمیل پرسشنامه ها و حذف موارد ناقص، براساس مصرف یا عدم مصرف مواد، آزمودنی ها به دو گروه ملاک (38 نفر) و گواه (36 نفر) طبقه بندی شدند. یافته‌ها: مقایسه پاسخ های دو گروه ملاک و گواه نشان داد که 52 گویه از روایی مناسب برخوردارند. پس از بررسی همسانی درونی مقیاس، 43 گویه با ضریب آلفای کرونباخ 0/95 باقی ماندند که در نقطه برش 99، نوجوانان دارای نگرش مثبت و منفی به اعتیاد را با حساسیت %93/1 و اختصاصیت %96/3 از هم تفکیک می‌کنند. نتیجه‌گیری: مقیاس سنجش نگرش نوجوانان نسبت به اعتیاد، معتاد و مواد مخدر که طی این پژوهش طراحی و بررسی گردید، از ویژگیهای روانسنجانه قابل قبول برای بکارگیری در جمعیت نوجوانان ایرانی برخوردار است.

Title: مقایسه روش‌های فورومتری کاور تست و مادوکس راد در فواصل دور و نزدیک

http://rehabilitationj.uswr.ac.ir/article-1-12-fa.html

هدف: در این تحقیق دو روش کاورتست و مادوکس راد (میله مادوکس) مورد مقایسه قرارگرفته است. این آزمونها جهت اندازه‌گیری هتروفوریا و ارزیابی دید دوچشمی در سندرم‌های دید دوچشمی مختلف مورد استفاده قرار می‌گیرند. روش بررسی: مطالعه حاضر به روش تحلیلی مقایسه‌ا‌ی و بررسی تستها می‌باشد. کاورتست آزمون استاندارد در زمینه بررسی حرکتی دید دوچشمی است. جامعه مورد مطالعه، دانشجویان دانشکده علوم توانبخشی بودند که به روش غیر احتمالی انتخاب شدند. این افراد بین 23- 18 سال و 123 نفر مؤنث و 79 نفر مذکر بودند. آزمونها بطور تصادفی و cross-over روی افراد در دو فاصله دور (m6) و نزدیک (cm40) انجام گرفت. عدم وجود اختلالات مؤثر بر هتروفوریا از شرایط انتخاب افراد در نظر گرفته شد. سپس نتایج ثبت شده مورد تجزیه و تحلیل آماری قرار گرفت. یافته‌ها: نتایج آماری نشان دهنده تمایل چشمها به اگزوفوریا (0/3- پریزم دیوپتر) در کاورتست و تمایل به ایزوفوریا در مادوکس راد (0/3 پریزم دیوپتر) در فاصله دور می‌باشد. این تفاوت به طور معنی داری مشاهده می‌شود (P<0/001). نتایج آزمونها در فاصله نزدیک بسیار مشابه بود (3 پریزم اگزوفوریا). نتیجه‌گیری: در این دو آزمون دو تفاوت اساسی سطح گسستگی (Dissociation) و سطح تحریک تطابقی وجود دارد. به نظر می‌رسد اختلاف مشاهده شده در دور تنها بعلت تفاوت سطح دیسوسیشن در این دو تست می‌باشد. این شرایط باعث عدم تفاوت معنی‌دار نتایج در نزدیک شده است زیرا اثر عدم فعالیت تطابقی با عدم دیسوسیشن کامل اثر یکدیگر را خنثی نموده‌اند. لذا برای بیماران مبتلا به اختلالات تطابقی و ورژنسی استفاده از مادوکس‌ رد در فاصله نزدیک مفید نیست.

Title: حوزه‌‌های بیوانرژتیک و مکانیسم تأثیرات زیستی

http://rehabilitationj.uswr.ac.ir/article-1-16-fa.html

توجه فزاینده عموم مردم به طب مکمل و جایگزین (CAM) دانشمندان را بر آن داشته است تا برای یافتن مکانیسم اثربخشی آنها تحقیقات گسترده‌ای انجام دهند. بعضی از این روش‌ها از قبیل یوگا، طب سوزنی و بیوفیلد تراپی‌ها بر حوزه‌های انرژی زیستی که درون و اطراف بدن انسان را فراگرفته است تأثیر می‌گذارند. بر اساس قوانین فیزیک، جریان‌یافتن الکتریسیته در بافتهای بدن، همانند آنچه که در قلب و مغز اتفاق می‌افتد، باعث تولید حوزه‌های بیومغناطیسی گردیده و حرکت ذرات باردار در بدن، همانند یونها و رادیکالهای آزاد، باعث تولید حوزه‌های الکترومغناطیسی می‌شود. تحقیقاتی که با کمک مغناطیس‌سنج‌های بسیار حساس صورت گرفته است، حوزه‌های بیومغناطیسی را ثبت و اندازه‌گیری نموده و نشان می‌دهد که تمرکز فکری و آرامش ذهنی، تا حدود 1000 برابر شدت آنها را تشدید می کند. این حوزه‌های بیومغناطیسی می تواند منشاء تأثیرات درمانی برخی از درمانهای مکمل و جایگزین مانند لمس درمانی باشد. از طرف دیگراستفاده از حوزه‌های الکتریکی، مغناطیسی و الکترومغناطیسی خارجی، در پزشکی کاربردهای شناخته شده‌ای دارند، مانند استفاده از حوزه‌های مغناطیسی در MRI و انواع درمان با حوزه‌های الکترومغناطیس نوساندار (PEMF تراپی). تحقیقات جدید در زمینه بیومغناطیس و مگنتوبیولوژی، بین روشهای به ظاهر متفاوتی که در طب رایج و طب مکمل به کار می‌رود، هم از بعد تأثیرات و هم از بعد مکانیسم، ارتباط برقرار می‌نماید. مقاله حاضر برخی از جدیدترین یافته‌های موجود را در زمینه حوزه‌های بیولوژیک و بیوانرژتیک و تأثیرات زیستی آنها، مرور نموده است.

Title: پرستاری بازتوانی بیمار محور برای افراد سالمند با مشکل دمانس

http://rehabilitationj.uswr.ac.ir/article-1-17-fa.html

با توجه به افزایش جمعیت سالمندان، نیاز مالی، بهداشتی و رفاهی آنان نیز افزایش می‌یابد. با توجه به اینکه اختلالات در سالمندان غالباً از نظر علائم، بیماریزایی و فیزیوپاتولوژی با جوانان متفاوت می‌باشد، لذا توجه بیشتری را می‌طلبد. دمانس پس از آرتریت موجب بیشترین ناتوانی در سالمندان می‌گردد. دمانس سندرمی است که اختلال شناختی بدون اختلال در سطح هوشیاری ایجاد می‌کند. همچنین منجر به ضعف حافظه، اشکال در محاسبه، حواس‌پرتی، تغییرات خلق و عاطفه، اختلال در قضاوت و تفکر انتزاعی، کاهش مهارت‌های زبانی و اختلال در جهت‌یابی می‌شود. از انواع دمانس می‌توان به دمانس آلزایمر و دمانس ناشی از بیماریهای پیک، ژاکوب ـ کروتسفلد، هانتینگتون، هیدروسفالی با فشار طبیعی و پارکینسون اشاره کرد. درمان شامل: درمان زمینه‌ای و همزمان مانند فشار خون بالا، درمان طبی مانند: استفاده از داروهای آنتی‌کولین استراز و برنامه بازتوانی طرح‌ریزی شده می‌باشد. همراه کردن اصول مراقبتی فردمحور در بازتوانی سالمندان با اختلال دمانس و تمرکز بر آن، اساس سازماندهی خدمات و ارائه مراقبتهای پرستاری می‌باشد.
